# Charting Progress: Clinical Outcomes and the Learning Curve of Robotic Cholecystectomy in Indian Surgical Practice

**DOI:** 10.7759/cureus.96399

**Published:** 2025-11-09

**Authors:** Atul N.C. Peters, Yogesh Gautam, Atul Wadhwa, Shalabh Agarwal

**Affiliations:** 1 Department of Bariatric, Minimal Access, and General Surgery, Max Smart Super Speciality Hospital, Saket, Delhi, IND; 2 Department of General Surgery, Max Smart Super Speciality Hospital, Saket, Delhi, IND

**Keywords:** cholecystectomy, learning curve, minimally invasive surgery, robotic-assisted surgery, robotic cholecystectomy

## Abstract

Background

With the growing use of robotic technology, robotic cholecystectomy (RC) is becoming more common. However, the effect of the learning curve on clinical outcomes in India is yet to be studied. This study addresses that gap by comparing short-term outcomes between two RC cohorts.

Methods

This study was conducted at a tertiary care center in India, involving 63 patients who underwent RC using the da Vinci Xi system. The patients were divided into two cohorts: cohort 1 included the first 31 cases, and cohort 2 comprised the next 32 cases, based on the learning curve. We evaluated short-term clinical outcomes both preoperatively and postoperatively.

Results

Patients undergoing RC were relatively young (mean age 45.10 ± 12.73 years) and predominantly female (68.25%). The average body mass index (BMI) was 30.67 ± 5.80 kg/m². The mean operative time of the study population was 37.16 ± 15.45 minutes, with port placement, docking, and console times averaging 1.03 ± 0.23, 0.81 ± 0.41, and 44.20 ± 18.66 minutes, respectively. Only one intraoperative complication (1.58%) occurred, and no conversions to open surgery were required. Postoperatively, there were no complications, bile duct injuries, or surgical site infections. The mean hospital stay was 1.90 ± 1.14 days. The pain scores decreased significantly over time at 6, 24, and 48 hours, and 7 days postoperatively, with no pain reported by day 14. Patients used an average of 2.65 ± 0.63 analgesics over 4.13 ± 1.55 days. Quality of life (QoL) scores improved steadily on postoperative days 3, 14, and 30, with no readmissions or disease recurrence. Cohort 2 showed a significantly reduced docking time (0.70 ± 0.25 vs. 0.93 ± 0.50 minutes, p=0.0269), indicating greater efficiency with experience.

Conclusion

RC is a feasible and effective option in India, offering improved short-term outcomes, faster recovery, and a learning curve of around 40 cases for experienced laparoscopic surgeons.

## Introduction

Acute cholecystitis is an inflammation of the gallbladder, most often caused by a blockage in the biliary tract or inadequate gallbladder emptying [1]. Gallstones affect 10-15% of the global population, with acute cholecystitis often serving as the first clinical manifestation in a similar proportion of these cases [2-3]. In the United States alone, approximately 200,000 cases of acute cholecystitis are reported each year [4].

Cholecystectomy is one of the most frequently performed surgeries worldwide for treating symptomatic or benign gallstones [4]. Since the mid-1990s, laparoscopic cholecystectomy (LC) has become the standard approach, offering benefits over open surgery such as shorter hospital stays, lower morbidity, and reduced mortality [5-6]. Despite its widespread use, there is still room for improvement in clinical outcomes [3]. Innovations such as robotic surgery have been developed to further reduce invasiveness and improve surgical precision [7-8]. Robot-assisted surgery (RAS) has gained steady traction in both benign and oncologic procedures since the early 2000s [9], with its use in benign surgeries such as cholecystectomy beginning in the mid to late 2000s [6]. On March 13, 1997, Himpens and Cadiere performed the first robotic cholecystectomy (RC) in a 72-year-old obese patient with the da Vinci system. RAS allows for single or multiple small incisions, with the surgeon operating remotely from a console [10]. Compared to open surgery, robotic procedures offer better outcomes in terms of hospital stay, complications, and mortality [11], especially in certain contexts, and may be even less invasive than laparoscopic surgery due to reduced tissue damage and inflammation [6]. However, evidence comparing RAS and laparoscopic approaches remains mixed regarding outcomes and cost-effectiveness [11]. Still, lower mortality rates per 10,000 procedures suggest that RAS could be a strong alternative to LC [6]. Several studies across surgical specialties have examined the learning curves associated with various robotic procedures, including the setup and docking process using the traditional da Vinci multiport system [12]. In recent years, RC has become increasingly common with the growing integration of robotic technology in surgical practice. With proper training of both the surgeon and the surgical team and with an increasing number of cases, the complexity of the operations can be increased and outcomes can be improved. However, the influence of the learning curve on clinical outcomes in the Indian context remains unreported. This study seeks to address that gap by evaluating and comparing short-term perioperative and postoperative clinical outcomes across different cohorts of RC cases. To the best of our knowledge, this represents the first analysis of the RC learning curve within an Indian healthcare setting.

## Materials and methods

The retrospective study was conducted at a tertiary care center in New Delhi, India. A review of medical records was conducted for consecutive patients who underwent non-complex RC between October 2023 and January 2025. Of these, the first 31 cases (cohort 1) were performed between October 2023 and August 2024, while the subsequent 32 cases (cohort 2) were operated on between September 2024 and January 2025. Non-complex RC was defined as elective cholecystectomy for benign indications, including simple gallstones, acute calculous cholecystitis, and small polyps. Preoperative imaging confirmed the absence of distant metastases or peritoneal disease. Cases with significant adhesions, distorted anatomy, or prior complex abdominal surgeries were excluded. In this context, distorted anatomy refers to the distortion around Calot’s triangle caused by inflammation and scarring, which made dissection challenging. The specific duration of adhesiolysis was not used to define significant adhesions, and, therefore, a time-based criterion was not considered. Cholecystectomy cases performed for malignant conditions or benign indications, such as emergent cholecystectomy for any benign indication and emergent or elective cholecystectomy for Tokyo grade II or III acute cholecystitis, porcelain gallbladder, large/atypical gall bladder polyp, were excluded from the study. Baseline characteristics, including age, sex, BMI, comorbidities, and the indication for cholecystectomy, were documented. Intraoperative data such as operative time, conversions, complications, and analgesic use were collected. Postoperative variables, comprising length of hospital stay, complications, analgesic use, pain scores, QoL scores, and rates of readmission and reoperation, were extracted from medical records. Pain was assessed using the 0 to 10 Numerical Rating Scale (NRS), where a score of “0” represents no pain and “10” represents the worst pain imaginable. The NRS is a single-question tool that is simple to administer, easy to understand, reproducible, and sensitive to small changes in pain levels. NRS scores were recorded at 6 hours, 24 hours, 48 hours, 7 days, and 14 days following surgery. In addition to NRS scores, data on the number of analgesics taken during the postoperative period and the duration of analgesic use were also documented. QoL was assessed using the EuroQol 5-Dimension 3-Level classification system (EQ-5D-3L) and was recorded on postoperative days 3, 7, 14, and 30 [13]. The EQ-5D-3L descriptive system comprises five dimensions: mobility, self-care, usual activities, pain/discomfort, and anxiety/depression. Each dimension is rated on three levels: no problems, some problems, and extreme problems. Additionally, the EQ visual analog scale (EQ-VAS) was used to measure the patient's self-rated health on a scale from 0 to 100, with 0 indicating the worst imaginable health state and 100 indicating the best imaginable health state. Data extraction and pain/QoL assessments were performed uniformly across all patients to maintain consistency and reduce measurement bias. The study was conducted following the ethical principles outlined in the latest version of the Declaration of Helsinki and adhered to applicable Good Clinical Practice (GCP) guidelines. Ethical approval for the study was obtained from the Institutional Ethics Committee under letter number BHR/RS/MSSSH/GMHRCMS/MHEC/BMAGS/25-03 dated July 14, 2025.

Robotic surgeries were performed using the Da Vinci Xi Surgical System (Intuitive Surgical, Sunnyvale, CA) following a standardized technique, following established guidelines from recognized surgical societies. The system provides high-definition, three-dimensional visualization and employs EndoWrist instruments with seven degrees of freedom, enhancing surgical precision and maneuverability. All procedures were conducted by a single, experienced surgeon. The port placement, docking, and surgical procedure were all performed by the same surgeon and his surgical team. The surgical learning curve was assessed using the risk-adjusted cumulative summation (CUSUM) method, which allows for sequential analysis of cases based on surrogate markers such as operative time, applied in our study. For each surgery, the CUSUM value was calculated chronologically by summing the differences between the individual operative time and the mean operative time across all cases. The CUSUM value for the first case was calculated as the difference between its operative time and the overall mean. For subsequent cases, each CUSUM value was obtained by adding the current case’s difference to the previous cumulative value. This iterative process generated a continuous CUSUM curve, highlighting trends in operative performance over time [14]. Quantitative variables were summarized using the arithmetic mean and standard deviation (SD), while categorical variables were reported as frequencies and percentages. Comparisons between categorical variables across study cohorts were made using Pearson’s chi-square test or Fisher’s exact test, as appropriate. Differences in means between cohorts were analyzed using two-sample t-tests. A two-sided p-value of <0.05 was considered statistically significant. All statistical analyses were performed using Stata version 16.0 (StataCorp LLC, College Station, TX).

## Results

Data from a total of 63 patients were included in this analysis. Of these, 68.25% were female and 31.75% were male, with a mean age of 45.10 ± 12.73 years. The average BMI was 30.67 ± 5.80 kg/m². The most common indication for surgery was cholelithiasis (74.6%), followed by acute calculous cholecystitis (23.81%) and gallbladder polyps (1.59%). Descriptive characteristics of the preoperative variables are summarized in Table 1.

**Table 1:** Descriptive characteristics of preoperative variables BMI, body mass index; CABG, coronary artery bypass graft; SD, standard deviation

Variable	N=63
Age, mean ± SD, year	45.10 ± 12.73
Sex, n (%)	
Female	43 (68.25)
Male	20 (31.75)
Weight, mean ± SD, kg	80.16 ± 15.75
Height, mean ± SD, cm	162.00 ± 8.65
BMI, mean ± SD, kg/m^2^	30.67 ± 5.80
Comorbidities, n (%)	
Hypothyroidism	15 (23.81)
Diabetes	10 (15.87)
Hypertension	10 (15.87)
Thalassemia	2 (3.17)
Obesity	2 (3.17)
CABG	2 (3.17)
Acute biliary pancreatitis	1 (1.59)
Chronic kidney disease	1 (1.59)
Epilepsy	1 (1.59)
Psoriasis	1 (1.59)
Rheumatoid arthritis	1 (1.59)
Traumatic headache	1 (1.59)
Previous abdominopelvic surgery, n (%)	15 (23.81)
Indication for cholecystectomy, n (%)	
Cholelithiasis	47 (74.60)
Acute calculous cholecystitis	15 (23.81)
Gallbladder polyps	1 (1.59)

Table 2 summarizes the intraoperative and postoperative variables of the study population.

**Table 2:** Intraoperative and postoperative variables of the study population QoL, quality of life; SD, standard deviation

Intraoperative variables	N=63
Operative time, mean ± SD, min	37.16 ± 15.45
Port placement time, mean ± SD, min	1.03 ± 0.23
Docking time, mean ± SD, min	0.81 ± 0.41
Length of stay, mean ± SD, days	1.90 ± 1.14
Ports, n (%)	
3	1 (1.59)
3+1	58 (92.06)
3+2	4 (6.35)
Conversion, n (%)	0 (0)
Intraoperative complications, n (%)	1 (1.59)
Postoperative complications before discharge, n (%)	0 (0)
Bile duct injury, n (%)	0 (0)
Surgical site infection, n (%)	0 (0)
Number of analgesics used in a day, mean ± SD	2.65 ± 0.63
Type of drugs used, n (%)	
Diclofenac	60 (95.24)
Paracetamol	58 (92.06)
Buscopan	33 (52.38)
Tramadol	5 (7.94)
Tapentadol	1 (1.59)
Length of analgesics use, mean ± SD, days	4.13 ± 1.55
Pain score after surgery, mean ± SD	
6 hours	2.47 ± 1.63
24 hours	0.87 ± 1.18
48 hours	0.27 ± 0.68
7 days	0.02 ± 0.13
14 days	0.0
QoL score after surgery, mean ± SD	
Day 3	93.98 ± 9.56
Day 7	98.93 ± 3.69
Day 14	99.67 ± 1.80
Day 30	99.66 ± 1.82
Postoperative complications after discharge, n (%)	0 (0)
Re-admission, n (%)	0 (0)
Recurrence, n (%)	0 (0)

The mean operative time was 37.16 ± 15.45 minutes, with a mean port placement time of 1.03 ± 0.23 minutes and docking time of 0.81 ± 0.41 minutes. The average length of hospital stay was 1.90 ± 1.14 days. There were no conversions to open surgery, and only one intraoperative complication was reported. No postoperative complications, bile duct injuries, or surgical site infections were observed. Pain scores demonstrated a marked decline at 6 hours, 24 hours, 48 hours, and 7 days postoperatively, with patients reporting no pain by day 14. The mean number of analgesics used was 2.65 ± 0.63, and the average duration of analgesic use was 4.13 ± 1.55 days. QoL scores showed progressive improvement on postoperative days 3, 14, and 30. There were no cases of readmission or disease recurrence during the study period.

Table 3 compares the preoperative, intraoperative, and postoperative variables between the two study cohorts: the first 31 cases (cohort 1) and the subsequent 32 cases (cohort 2).

**Table 3:** Comparison of preoperative, intraoperative, and postoperative variables between the study cohorts *Statistically significant SD, standard deviation

Variables	Cohort 1 (first 31 cases)	Cohort 2 (subsequent 32 cases)	t-Value/chi-square value	p-Value
Operative time, mean ± SD, min	40.78 ± 17.90	33.66 ± 12.20	1.8506	0.0691
Port placement time, mean ± SD, min	1.06 ± 0.25	1.00 ± 0.22	1.0890	0.2804
Docking time, mean ± SD, min	0.93 ± 0.50	0.70 ± 0.25	2.2675	0.0269*
Conversion, n (%)	0 (0)	0 (0)	-	-
Intraoperative complications, n (%)	0 (0)	1 (3.13)	0.9844	0.321
Postoperative complications before discharge, n (%)	0 (0)	0 (0)	-	-
Bile duct injury, n (%)	0 (0)	0 (0)	-	-
Surgical site infection, n (%)	0 (0)	0 (0)	-	-
Postoperative complications after discharge, n (%)	0 (0)	0 (0)	-	-
Re-admission, n (%)	0 (0)	0 (0)	-	-
Recurrence, n (%)	0 (0)	0 (0)	-	-
Indication for cholecystectomy, n (%)				
Acute calculous cholecystitis	4 (12.90)	6 (18.75)	-	0.5255
Calculous cholecystitis	1 (3.23)	4 (12.50)	-	0.1734
Cholelithiasis	25 (80.65)	22 (68.75)	-	0.2782
Gallbladder polyps	1 (3.23)	0	-	0.3058

No statistically significant differences were observed between the cohorts in terms of operative time, port placement time, intraoperative complications, or the indications for cholecystectomy. However, a significant reduction in docking time was noted in cohort 2 compared to cohort 1 (0.70 ± 0.25 vs. 0.93 ± 0.50 minutes, p=0.0269), suggesting increased procedural efficiency with experience.

**Figure 1:**
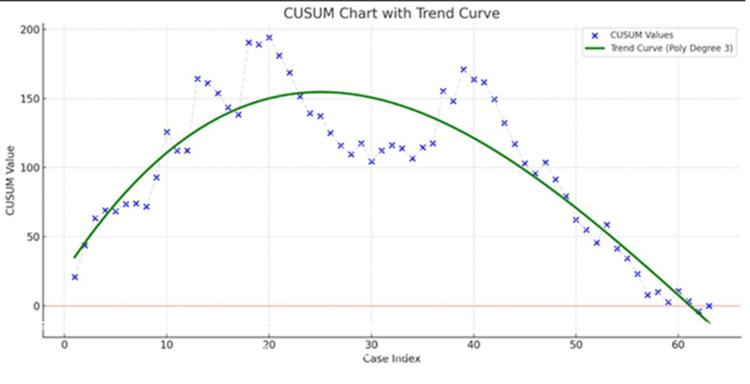
The learning curve of robot-assisted cholecystectomy

The CUSUM analysis of robot-assisted cholecystectomy highlighted three phases in the surgeon’s performance progression (Figure 1).

In the initial learning phase (cases 1-18), CUSUM values rose from 20.84 to 190.32, reflecting early adaptation to instruments, spatial orientation, and procedural steps, typical of the initial technical learning curve. During the stabilization phase (cases 19-38), values plateaued between 150 and 190, indicating improved consistency with some variability as skills were consolidated. From case 39 onward, a sustained decline from 103 to -3.72 marked the attainment of surgical proficiency, with consistent outcomes and minimal deviation from expected standards.

## Discussion

Cholelithiasis is a leading cause of surgical admissions, with cholecystectomy being one of the most frequently performed general surgeries worldwide [15]. Acute cases account for 20-40% of procedures, while 60-80% are elective [16]. Minimally invasive surgery (MIS), introduced in the 1980s and widely adopted by the 1990s, has transformed gallbladder surgery [17]. Cholecystectomy was among the first procedures to shift from open to MIS [8], and recent advancements in robot-assisted techniques have accelerated progress across surgical disciplines [11]. RC saw a 37-fold increase in use from 2010 to 2019, rising from 0.1% to 5.2% of cases, while open cholecystectomy rates declined from 24.4% to 17.0% in the same period [18].

Earlier studies have highlighted that RAS may address limitations of LC by improving tactile feedback, reducing surgeon fatigue, enhancing visualization, precise instrument control, 3D visualization, and fewer collisions, all of which contribute to the safer execution of complex procedures [3,11,19]. Surgeons are increasingly adopting RC for its perceived safety, particularly in the context of acute cholecystitis, common bile duct exploration, and patients with previous upper abdominal surgeries, where there are significant improvements to be made with respect to patient outcomes. Secondly, it is seen as an opportunity to develop robotic skills through a familiar procedure [18]. However, in India, its uptake has been slower compared to Western countries, with significant growth only beginning in the late 2010s [3]. Also, the financial aspects of running a robotic program have to be considered. The clinical effectiveness of RC in the Indian context remains insufficiently studied, and the impact of the learning curve on patient outcomes is still debated [9]. This study seeks to evaluate and compare clinical outcomes across various stages of the RC learning curve in a retrospective cohort in an Indian setting.

In this study, the primary indication for surgery was cholelithiasis (74.6%), followed by acute calculous cholecystitis (23.81%) and gallbladder polyps (1.59%). These findings are consistent with a previous Indian study comparing RC and LC, where symptomatic cholelithiasis was the most common preoperative indication in the RC group (52.5%) [3].

The intraoperative and postoperative indices are crucial indicators of surgical safety. In this study, the mean operative time was 37.16 ± 15.45 minutes, with a mean port placement time of 1.03 ± 0.23 minutes, docking time of 0.81 ± 0.41 minutes, and console time of 44.20 ± 18.66 minutes. These results align with an Indian study comparing RC and LC, which reported a mean operative time of 40 ± 9.78 minutes in the RC group [3]. Additionally, a meta-analysis revealed a statistically significant increase in operative duration for single-port RC (weighted mean difference [WMD] = 26.67, 95% CI 14.99 to 38.34, I² = 93%, p<0.00001) compared to single-port LC [8]. The relatively lower operative time in our study may be attributed to a shorter docking time in cohort 2, which is consistent with earlier findings suggesting that reduced docking time contributed to shorter operative times in RC [3]. Another Indian study reported a comparatively shorter mean operative time of 25.19 minutes (range: 10-87 minutes) for procedures performed using a robotic approach [20]. The average times for draping and robotic system setup were 6.44 minutes (range: 4-13 minutes) and 6.64 minutes (range: 3-15 minutes), respectively. A gradual decline in operative, draping, and setup durations was noted for 100 consecutive cases [21]. Our study demonstrates a shorter robot setup time, including port placement and docking.

Intraoperative complications are critical for assessing the risk profiles of surgical procedures and understanding their associated dangers [8]. In our study, only one (1.59%) patient experienced an intraoperative complication. An Indian study comparing RC and LC reported no intraoperative complications in the robotic group [3]. Similarly, another Indian study showed no incidents of intraoperative complications, such as bleeding or common bile duct injury, in either the RC or LC group (0% vs. 0%) [9]. Bile duct injury rates following RC tend to decrease with growing surgical experience, and it is estimated that between 300 and 450 RC cases are required to achieve bile duct injury rates comparable to those of conventional LC [18]. Conversion rates are an important indicator of surgical technique, as they reflect the surgeon’s judgment and may reassure both the patient and the surgeon when lower rates are observed [8]. In our study, there were no conversions to open surgery, which aligns with existing literature. A previous study also reported no conversions to open surgery [8]. Similarly, another Indian study comparing RC and LC showed no conversions in either group (0% vs. 0%) [3,9]. Additionally, a systematic review by Straatman et al. reported a significantly lower risk of conversion in the robotic arm [15].

Regarding postoperative outcomes, our study found that the average length of hospital stay in the RC group was 1.90 ± 1.14 days. This is consistent with a previous Indian study, which reported a shorter hospital stay in the RC group (1.08 ± 0.26 days) [3]. Another Indian study also found the mean length of hospital stay to be one day for both RC and LC groups [9]. However, a meta-analysis study concluded a statistically significant decrease in the duration of hospitalization of approximately half a day in the single-port RC compared to single-port LC (WMD = −0.52 [95% CI −0.89, −0.14], I^2^ = 0%, P [heterogeneity]=0.52, P [overall]=0.007) [8]. Shorter hospital stays are often associated with more efficient care [8], and cholecystectomy is increasingly performed as a day-care surgery, except for patients admitted for other medical reasons, which can also affect their length of stay [11]. Our findings on postoperative complications, bile duct injuries, and surgical site infections are consistent with a previous Indian study comparing RC and LC, which reported no postoperative complications, bile duct injuries, surgical site infections, or mortality at 30 days post-surgery [3]. Another Indian study also showed no postoperative complications in the RC group (0% vs. 0%) [9]. A meta-analysis of seven randomized controlled trials suggested that the use of subcuticular sutures or staples could help prevent surgical site infections [22]. In our study, there were no cases of readmission or disease recurrence during the follow-up period, which aligns with several studies reporting no readmissions, mortality, or re-operations in the RC group [3,9].

Our findings on pain scores are supported by an Indian study that showed a decrease in pain score from 1.15 ± 0.95 on day 4 to 0.45 ± 0.96 on day 7 post-surgery, with the RC group having a median visual analog scale (VAS) pain score of 0 on postoperative days 2 and 7 [3]. Similarly, Lee et al. reported lower pain scores in the RC group on postoperative days 1, 2, and 7 when comparing single-port RC to multiport LC [22]. Another study showed that the mean pain score 24 hours after surgery was 1.78 ± 0.68 in the robotic group and 3.3 ± 1.2 in the laparoscopic group (p<0.001) [9]. Cho et al. also found that pain scores at 2 (p<0.04), 4 (p<0.02), and 8 (p<0.02) hours after surgery were significantly lower in the RC group compared to the LC group [23]. Lower pain levels may be due to smaller incisions and the use of robotic arms during the procedure, which, according to a previous study, reduce torque applied to the abdominal wall, contributing to less pain in the RC group [3]. Additionally, the port positions in RC eliminate the need for an epigastric port, which may also explain the lower pain levels in the RC group [3]. In our study, the mean number of analgesics used was 2.65 ± 0.63, and the average duration of analgesic use was 4.13 ± 1.55 days. A prior study found that no patients in the robotic group required opioid analgesics [9].

In this study, we found no statistically significant differences between the two study cohorts (based on the learning curve) in terms of operative time, port placement time, length of hospital stay, intraoperative complications, or indications for cholecystectomy. However, a significant reduction in docking time was observed in cohort 2 compared to cohort 1 (0.70 ± 0.25 vs. 0.93 ± 0.50 minutes, p=0.0269), indicating increased procedural efficiency with experience. The first Indian case series on RC by Chowbey et al. demonstrated a consistent reduction in operating time as the surgical team progressed through their learning curve, particularly when a single console was shared by both the attending surgeon and the resident trainee [20]. A comparison of the first and last 50 RC procedures showed significant improvements in workflow efficiency, including reduced times for draping, positioning, and console work [20]. Team coordination improved after around 30 cases, with minor adjustments in technique contributing to more streamlined procedures, enhanced ergonomics, greater precision, and better patient recovery [20]. Our observation of a sustained decline in CUSUM values from case 39 onward aligns with the findings of this study, indicating the achievement of surgical proficiency, characterized by consistent outcomes and minimal deviation from expected performance standards. Additionally, previous studies have indicated that, during the learning phase, RC does not increase patient morbidity when compared to LC [9,15]. Another study highlighted that early docking training for all team members and maintaining a consistent team improved operating room efficiency over time [3]. The significant reduction in docking time observed in cohort 2 of our study further reinforces this observation, as our operative team was trained specifically and remained consistently the same for all procedures.

Strengths and limitations

All procedures were performed by a single experienced surgeon, ensuring consistency in surgical workflow and minimizing operator-related variability. However, the study has several limitations inherent to its retrospective, single-arm design. The primary surgeon possesses substantial experience in both multi-port and single-port laparoscopic techniques and currently serves as a mentor to many practicing surgeons across India. As a result, the outcomes observed may not be generalizable to all surgeons. Additionally, the surgeon was concurrently performing other robotic procedures during the study period, further contributing to their overall proficiency with the robotic platform. The reliance on existing medical records limited the ability to comprehensively evaluate long-term complications, as follow-up data were incomplete. Moreover, the retrospective nature of the study precluded a detailed cost-effectiveness analysis, which is essential for assessing the broader adoption of robotic surgery in the Indian healthcare context. Other key limitations include the relatively small sample size, the presence of potential confounders such as comorbidities and prior surgeries, and a short follow-up duration, all of which may affect the generalizability and robustness of the findings.

## Conclusions

In conclusion, the findings of this study suggest that RC offers potential advantages in perioperative and postoperative outcomes and is feasible within Indian settings. Additionally, the reduction in pain and faster recovery may enhance the QoL for patients undergoing RC. Furthermore, an experienced laparoscopic surgeon could achieve proficiency in RC after performing approximately 40 cases. However, the selection of patients and the complexity of cases may also influence operating time. These findings could potentially influence clinical practices regarding cholecystectomy treatment in India. However, additional long-term follow-up studies are needed to evaluate the sustained impact of the learning curve on patient outcomes. Future studies should thoroughly examine various indicators, including complexity scores, objective performance metrics, and video feedback.
